# Coral Bacterial-Core Abundance and Network Complexity as Proxies for Anthropogenic Pollution

**DOI:** 10.3389/fmicb.2018.00833

**Published:** 2018-04-27

**Authors:** Deborah C. A. Leite, Joana F. Salles, Emiliano N. Calderon, Clovis B. Castro, Adalto Bianchini, Joseane A. Marques, Jan Dirk van Elsas, Raquel S. Peixoto

**Affiliations:** ^1^Instituto de Microbiologia, Universidade Federal do Rio de Janeiro, Rio de Janeiro, Brazil; ^2^Groningen Institute for Evolutionary Life Sciences, University of Groningen, Groningen, Netherlands; ^3^Núcleo em Ecologia e Desenvolvimento Sócio-Ambiental de Macaé, Universidade Federal do Rio de Janeiro, Rio de Janeiro, Brazil; ^4^Instituto Coral Vivo, Santa Cruz Cabrália, Brazil; ^5^Museu Nacional, Universidade Federal do Rio de Janeiro, Rio de Janeiro, Brazil; ^6^Instituto de Ciências Biológicas, Universidade Federal do Rio Grande, Rio Grande, Brazil; ^7^Programa de Pós-Graduação em Oceanografia Biológica, Universidade Federal do Rio Grande, Rio Grande, Brazil; ^8^Instituto Museu Aquário Marinho do Rio de Janeiro-AquaRio – Rio de Janeiro Marine Aquarium Research Center, Rio de Janeiro, Brazil

**Keywords:** coral, core, *Mussismilia hispida*, pollution, proxies

## Abstract

Acclimatization via changes in the stable (core) or the variable microbial diversity and/or abundance is an important element in the adaptation of coral species to environmental changes. Here, we explored the spatial-temporal dynamics, diversity and interactions of variable and core bacterial populations associated with the coral *Mussismilia hispida* and the surrounding water. This survey was performed on five reefs along a transect from the coast (Reef 1) to offshore (Reef 5), representing a gradient of influence of the river mouth, for almost 12 months (4 sampling times), in the dry and rainy seasons. A clear increasing gradient of organic-pollution proxies (nitrogen content and fecal coliforms) was observed from Reef 1 to Reef 5, during both seasons, and was highest at the Buranhém River mouth (Reef 1). Conversely, a clear inverse gradient of the network analysis of the whole bacterial communities also revealed more-complex network relationships at Reef 5. Our data also indicated a higher relative abundance of members of the bacterial core, dominated by *Acinetobacter* sp., at Reef 5, and higher diversity of site-stable bacterial populations, likely related to the higher abundance of total coliforms and N content (proxies of sewage or organic pollution) at Reef 1, during the rainy season. Thus, the less “polluted” areas may show a more-complex network and a high relative abundance of members of the bacterial core (almost 97% in some cases), resulting in a more-homogeneous and well-established bacteriome among sites/samples, when the influence of the river is stronger (rainy seasons).

## Introduction

Host-bacteriome relationships and the effects of these interactions on the metaorganism development, ecology and evolution in coral reefs are currently under active study (Bosch and McFall-Ngai, 2011; Thompson et al., 2014; Webster and Reusch, 2017; Wegley Kelly et al., 2018). However, more information is required about the forces modulating this functional unit, the coral holobiont (Rohwer et al., 2002), and how the diversity and interactions among microbial members of the holobiont can contribute to or respond to environmental stressors (Kelly et al., 2014; Roder et al., 2015; van de Water et al., 2017).

The coral microbiome can be modulated by the host (Rosenberg et al., 2007a, 2009; Rosenberg and Zilber-Rosenberg, 2008), and is at the same time highly dynamic and closely affected by environmental conditions (Kelly et al., 2014; Roder et al., 2015; van de Water et al., 2017). This dynamic microbiome seems to be able to rapidly adapt (and evolve) in the face of environmental changes, through microbiome-mediated transgenerational acclimatization (MMTA) (Webster and Reusch, 2017). In this regard, a “healthy,” or beneficial (Peixoto et al., 2017) microbiome could, under stressful environmental conditions, safeguard the coral from the establishment of pathogens (Sweet and Bulling, 2017), thus protecting corals against dysbiotic processes (Webster and Reusch, 2017).

Considering the complex nature of the coral and its associated bacterial-microbiome relationships, the core bacteriome, i.e., the set of bacterial species shared among multiple microbial assemblages (Turnbaugh et al., 2007; Shade and Handelsman, 2012), as well the core dynamics, networks and their transmission to the coral offspring (Ainsworth et al., 2015; Kellogg et al., 2017; Leite et al., 2017, 2018; van de Water et al., 2017) represent a good target to simplify analyses and identify key players within a community. This selective approach focuses only on the persistent members of the bacteriome (Astudillo-García et al., 2017). In parallel, the transient bacteriome, shaped mostly by environmental sources and forces (van de Water et al., 2017), is likely associated with strategic mechanisms for the holobiont to quickly acclimate to changes in the environment (Webster and Reusch, 2017), and this part of the bacteriome is also a good target to explore. Knowledge of how the core (Ainsworth et al., 2015; Kellogg et al., 2017; Leite et al., 2017, 2018) and the transient communities are affected by local (e.g., oil and organic matter) and global (e.g., climate changes) impacts (Shade and Handelsman, 2012; Santos et al., 2014, 2015; Mustafa et al., 2016) and how this may affect the health of the metaorganism may provide valuable insights for subsequent studies of possible manipulation of beneficial microorganisms (Peixoto et al., 2017), as proposed by Shade and Handelsman (2012), Damjanovic et al. (2017), and Peixoto et al. (2017).

Our group has previously described the bacterial core associated with mucus of the coral *Mussismilia hispida* (Leite et al., 2018), showing that members of this core are present in different coral life stages and are transmitted to the coral offspring through their gametes (Leite et al., 2017). Here, we explored how the diversity and the interactions among members of bacterial core, and also the variable populations, can be affected by a gradient of space (representing also a gradient of anthropogenic influence, through the influence of the Buranhém River) and time. For this purpose, we carried out a survey of the bacteria associated with *M. hispida* colonies and the surrounding water, across five reef sites and four different sampling times. We also aimed to detect specific bacterial players associated with different locations (and the influence of the river) to explore possible local adaptations of the bacterial core.

## Materials and Methods

### Ethics Approval and Consent to Participate

Permission for sampling was obtained from the Brazilian Institute of Environment and Renewable Natural Resources (IBAMA)/Chico Mendes Institute for Biodiversity Conservation (ICMBio), permanent license number 16942-1, in accordance with Normative Instruction No. 03/2014 of the System of Authorization and Information on Biodiversity (SISBIO), and from local authorities of the Municipality Environmental Agency (SMMA), Porto Seguro, Bahia, Brazil. Permission for the microbial survey was obtained from CNPq (National Council for Scientific and Technological Development).

### Study Area and Sampling Procedures

Every 2–3 months between August 2013 and May 2014 (Rainy season 1: 21–27 August 2013; Rainy season 2: 3–7 December 2013; Dry season 1: 31–25 February 2014; Dry season 2: 20–24 May 2014) (**Supplementary Figure S1**), fragments of visually healthy *M. hispida* colonies (containing mucus, tissue and skeleton) and 1 L of the surrounding seawater (∼40 cm distant from the colonies) were sampled using sterilized bottles at five reefs located close to a protected marine area (“Parque Natural Municipal do Recife de Fora”) of Porto Seguro, Bahia, Brazil. Four of these reefs are located approximately 2 km from each other (Reef 1 [R1]: Itassepocu Reef – 16° 25.9′ 46.37″ S/039° 02′ 22.99″ W; Reef 2 [R2]: Pedra Carapindauba – 16° 25′ 29.32″ S/39° 01′ 19.42″ W; Reef 3 [R3]: Baixio Cerca – 16° 25′ 14.03″ S/39° 00′ 12.95″ W; Reef 4 [R4]: Recife de Fora-SW – 16° 25′ 12.91″ S/38° 58′ 58.80″ W; Reef 5 [R5]: Recife de Fora-NW – 16° 23′ 23.72″ S/38° 58′ 54.92″ W), starting 2 km off the mouth of the Buranhém River and ending at a patch of reef near the southeastern part of the Recife de Fora. Reef 5 is located in the northeastern area of the “Parque Natural Municipal do Recife de Fora,” theoretically away from the direct influence of the river mouth.

Coral sampling was performed in quadruplicate, so each colony served as a replicate. Seawater samples were also collected in four replicates at each site and were filtered through a 0.22-μm filter, using a standard vacuum-pump system (Prismatec 131B). All samples (coral fragments and filters) were immediately frozen in liquid nitrogen and stored at –80°C in the laboratory.

### DNA Extraction

Coral samples (containing mucus, tissue and skeleton) and seawater material scraped off the filters were homogenized, and the DNA was extracted using the PowerSoil^®^ DNA Isolation Kit (Mo Bio Laboratories, Carlsbad, CA, United States USA) following a modification of the method described by Sunagawa et al. (2010).

### Bacterial Community

PCR-DGGE was used to determine the bacterial community profile and to screen the samples, following exactly the same protocol described by Leite et al. (2017) (section Bacterial Profile). Accordingly, based on the bacterial profiles obtained, samples with different profiles were selected to perform the sequencing approach. The V4 variable region of the 16S rRNA gene from all samples was amplified using the primers 515F/806R (Caporaso et al., 2011) and paired-end (2 × 250 bp) sequencing was done at the Argonne National Laboratory, United States, in their Next Generation Sequencing Core on an Illumina Miseq, following the manufacturer’s guidelines. The QIIME 2 software package (version 2017.10)^1^ was used to process the raw sequence data (Bokulich et al., 2017). In brief, a total of 150,108 reads were obtained after demultiplexing with q2-DEMUX, with an average sequence length of 250 bp. The quality was filtered, replicated and chimera removed with q2-DADA2 (Callahan et al., 2016). Representative sequence sets for each DADA2 sequence variant were used for taxonomic classification.

The remaining high-quality sequences were binned into operational taxonomic units (OTUs) at 99% sequence identity using vSEARCH (Rognes et al., 2016). A representative sequence for each phylotype was aligned against the Greengenes database (DeSantis et al., 2006). Before further analysis, singletons, chloroplast plastids, mitochondria and archaeal sequences were removed from the dataset. For all OTU-based analyses, the original OTU table was rarified to a depth of 1,900 sequences per sample, to minimize the effects of sampling effort on the analysis. The QIIME 2 package was also used to generate weighted UniFrac distance matrices (Lozupone et al., 2006) and α-diversity metrics, including richness and diversity indices. All sequences were deposited in the NCBI Sequence Read Archive database with the accession number (SAMN08391548–SAMN08391636).

Here, we considered the “core” as a subset of bacterial taxa universally present in all samples (Turnbaugh et al., 2007; Shade and Handelsman, 2012). Considering that these microbes are widely distributed across bacteriomes, they could play key roles in an ecosystem (Turnbaugh et al., 2007; Shade and Handelsman, 2012). The coral core bacteriome was identified using QIIME (version 1.9.1) (Caporaso et al., 2010) and determined by plotting OTU abundance. It was represented by OTUs shared by at least 80% of the samples, considering four replicates of each sampling point and time, and by 100% of the samples considering each treatment (sampling time and point).

Network analyses were conducted on a subset of the 150 most abundant OTUs of coral bacteriomes from *M. hispida.* Significant correlations between OTUs with a minimum occurrence of 8 were determined using Pearson’s correlation and Spearman’s correlation (between -0.50 and 0.50, *p* < 0.05) using the CoNet app (Faust and Raes, 2012) in Cytoscape v.3.0.2 (Shannon et al., 2003).

A Venn diagram from site-stable bacterial populations (persistent OTUs found at each reef/sampling point) was obtained using the web-based tool InteractiVenn (Heberle et al., 2015).

### Physical and Chemical Analyses

Sediment samples (∼0.3 mg) were dried (60°C) and ground, and the percentages of N, C, and carbonate were determined via Costech EA-Delta XP IRMS. Seawater from each collection site (*n* = 4, 15 ml each) was filtered (0.45 μm), acidified (1% HNO_3_), protected from light with aluminum foil, and kept at -20°C until analysis. The dissolved organic carbon (DOC) concentration in seawater was determined in a Total Organic Carbon (TOC) analyzer (5050A, Shimadzu, Japan). Ion concentrations (Na, K, and Ca) were determined in seawater samples via flame photometry.

### Statistical Analyses

DGGE band profiles were digitalized and converted to data matrices using the Bionumerics v6.0 package (Applied Maths) according to the manufacturer’s instructions. The matrices were ordered by non-metric dimensional scaling (NMDS) (Kruskal, 1964; Mather, 1976) using a Bray-Curtis distance matrix. To analyze differences between the profiles and composition of the bacterial communities, we used NMDS with PRIMER6 (Kelly et al., 2015). The variation among different samples (time and location factors) was measured using permutational multivariate analysis of variance, PERMANOVA (Kelly et al., 2015), using PRIMER6 and the PERMANOVA+ extension (Anderson et al., 2008).

Estimates of α-diversity and β-diversity were based on an evenly rarified OTU abundance matrix. For β-diversity metrics, we analyzed the differences among the profiles and composition of bacterial communities, using a Principal coordinates analysis (PCoA), with a Bray-Curtis distance matrix with PRIMER6 (Kelly et al., 2015). To assess the variation among different samples, we used a permutational multivariate analysis of variance, PERMANOVA (Kelly et al., 2015) using PRIMER6 and the PERMANOVA+ extension (Anderson et al., 2008).

The OTUs associated with the bacterial family Enterobacteriaceae, i.e., potentially including coliforms, were used as a proxy for anthropogenic pollution.

Statistical differences of α-diversity matrices (observed OTUs; phylogenetic distance, PD; and the Chao index), nitrogen content, Enterobacteriaceae, core and variable OTUs, and site-stable bacterial populations (persistent OTUs found at each reef/sampling point) among the samples were determined using a Mann–Whitney test in Statistica software (v. 7.1). Pearson’s correlation was used to test for a relationship between nitrogen content and abundance of Enterobacteriaceae OTUs.

## Results

### Overall Water and Coral Bacterial Communities

Non-metric dimensional scaling of the 16S rRNA gene-based PCR-DGGE profile of bacterial communities from *M. hispida* colonies and the surrounding seawater showed that those communities were dynamic, changing in time and space (**Supplementary Figures S2, S3**). For all sampling times (rainy and dry seasons), the bacterial communities from the surrounding seawater differed among the sampling sites, according to the PERMANOVA (*F* = 9.1–28.0, *p* = 0.001) (**Supplementary Figure S2**). The same pattern was observed for the bacterial communities of *M. hispida* according to the PERMANOVA (*F* = 2.5–15.2, *p* = 0.001) (**Supplementary Figure S3**). However, the coral bacterial community from Reef 3 seemed to show an intermediate profile. Depending on the season, this community seemed to be more similar to the nearshore reefs, Reefs 1 and 2 (*p* > 0.05) (**Supplementary Figures S3B,C**), or to the offshore reefs, Reefs 4 and 5 (*p* > 0.05) (**Supplementary Figure S3A**). In contrast, the microbial communities from Reefs 4 and 5 differed significantly from Reef 1 at all sampling times (*p* < 0.05) (**Supplementary Figure S3**).

For the subsequent analysis of the bacterial diversity (Illumina Sequencing), Reef 1 (closest to the coast and to the Buranhém River mouth), Reef 3 (midway between Reefs 1 and 5) and Reef 5 (farthest from the Buranhém River mouth) were chosen, based on the DGGE profiles observed at the four sampling times.

**FIGURE 1:**
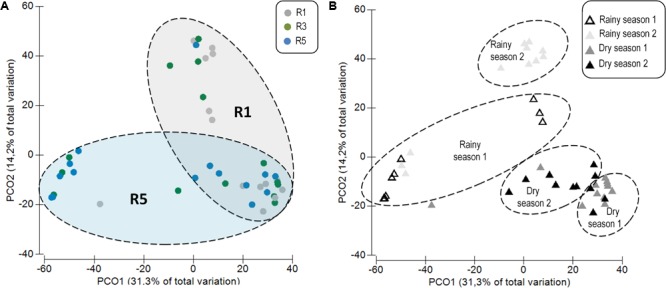
Principal coordinates analysis (PCoA) (weighted UniFrac) based on the distance matrix for operational taxonomic units (OTUs) showing differences among the *Mussismilia hispida* bacteriome at **(A)** different sampling locations (R1, R3, and R5) and **(B)** over time (rainy and dry seasons). **(A)** Circles correspond to the different sites, and **(B)** triangles correspond to the temporal variations of the bacteriome. Dotted contours indicate the groups obtained by comparisons with PERMANOVA, considering the effect of **(A)** location and **(B)** time on the *M. hispida* bacteriome (*p* = 0.01).

Principal coordinates analysis based on sequencing data revealed two main distinct groups, showing that the bacterial communities associated with *M. hispida* coral and the surrounding seawater were significantly different from each other (PERMANOVA: *F* = 42.50, *p* < 0.001) (**Supplementary Figure S4A**). In addition to differences in composition, the seawater bacterial communities were three times richer than those found in association with the coral (Tukey test, *F* = 247.8, *p* < 0.001) (**Supplementary Figure S4B**). According to sequencing data, *M. hispida* bacterial communities were dynamic (**Figure 1**), varying mainly according to the sampling location (Two-way PERMANOVA, *F* = 1.8, *p* = 0.045). Additionally, only samples from Reefs 1 and 5 showed distinct profiles (*F* = 1.7, *p* = 0.014). The coral bacterial communities sampled at Reef 3 could not be discriminated from samples from Reef 1 (*F* = 1.1, *p* = 0.28) or Reef 5 (*F* = 1.1, *p* = 0.21). The bacterial communities associated with *M. hispida* also varied significantly over the four sampling times (Two-way PERMANOVA, *F* = 6.3, *p* = 0.001) (**Figure 1B**). No significant differences were observed between the location and sampling period for any of the diversity indexes tested (**Table 1**) (Mann–Whitney test, *p* < 0.05). In addition, the rarefaction curves from all samples reached a plateau (**Supplementary Figure S5**).

**Table 1:** Overview of alpha diversity metrics (mean ± standard deviation) of *Mussismilia hispida* bacteriomes (*n* = 4).

Location	Period	Phylogenetic distance	Chao	OTUs numbers		
				Total	Site-stable	Core
R1	Rainy season 1	6.86 ± 0.23	56.00 ± 2.00^b^	56.00 ± 5.00^b^	9	6
	Rainy season 2	9.99 ± 1.52	101.55 ± 22.79^a^	94.25 ± 14.45^a^		
	Dry season 1	8.05 ± 2.86	61.40 ± 19.44^b^	57.50 ± 22.43^b^		
	Dry season 2	7.47 ± 1.20	57.48 ± 8.04^b^	50.93 ± 8.29^b^		
R3	Rainy season 1	6.02 ± 1.63	50.93 ± 16.77	69.15 ± 17.91	5	
	Rainy season 2	6.67 ± 1.68	69.15 ± 26.27	53.50 ± 24.51		
	Dry season 1	8.10 ± 2.30	53.50 ± 14.79	56.03 ± 14.91		
	Dry season 2	7.20 ± 2.22	56.03 ± 16.41	32.20 ± 17.44		
R5	Rainy season 1	3.83 ± 0.87	32.20 ± 9.76	64.23 ± 6.54	4	
	Rainy season 2	6.21 ± 1.31	64.23 ± 14.96	53.00 ± 9.82		
	Dry season 1	5.86 ± 1.62	39.63 ± 12.95	39.50 ± 13.09		
	Dry season 2	9.48 ± 3.32	77.99 ± 36.90	76.75 ± 35.50		

*OTUs, operational taxonomic units. Different letters indicate significant difference between samples.*

The OTUs associated with the family Enterobacteriaceae showed a significant gradient in abundance, with the highest prevalence at Reef 1, closest to the Buranhém River mouth (Mann–Whitney test, *p* < 0.05) (**Supplementary Figure S6A**).

The functional correlation between the abundances of Enterobacteriaceae OTUs from the coral microbiome and the seawater N content was also positive (Pearson′s r = 0.165) (**Supplementary Figure S6B**), showing a significant gradient of the N content, where Reef 1 was again the site of the highest concentration. No significant differences in salinity, C, carbonate, and DOC were detected among the different sampling sites (**Supplementary Table S1**).

### Coral Core and Site-Stable Bacteriomes

**FIGURE 2:**
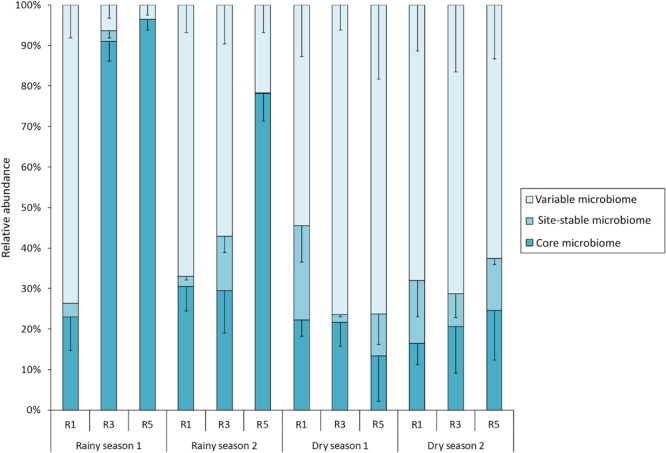
Contributions of variable, local, and core bacteriomes to the overall bacterial community of *M. hispida* at different sampling locations (R1, R3, and R5) and over time (rainy and dry seasons) (*n* = 4).

In order to explore the dynamics of variable and permanent bacterial populations associated with *M. hispida*, the core (OTUs shared among all sampling times and points), the site-stable bacterial populations (persistent OTUs found at each reef/sampling point) and the variable bacterial populations (other OTUs, calculated by subtracting the core and the site-stable bacterial populations) were evaluated (**Figure 2**). During rainy seasons, at Reef 5, the coral bacteriome was composed of a higher proportion (relative abundance) of the bacterial core (74–96%) (Mann–Whitney test, *p* < 0.05). Significant differences among the site-stable bacterial populations (persistent OTUs found at each reef/sampling point) of the reefs were observed only during rainy season 2. Therefore, there was a proportionately higher predominance of the site-stable bacterial populations at R3 (15%), followed by R1 (5%) and R5 (<1%), respectively (Mann–Whitney test, *p* < 0.05). On the other hand, Reef 3 showed different proportions of the variable and core bacteriomes (**Figure 2**) depending on the sampling period.

During dry seasons, there were no significant differences in the proportions of the variable or core bacterial microbiomes among the reefs. However, in dry season 1, Reef 1 showed a higher proportion of site-stable bacterial populations compared to Reefs 3 and 5 (Mann–Whitney test, *p* < 0.05) (**Figure 2**).

**FIGURE 3:**
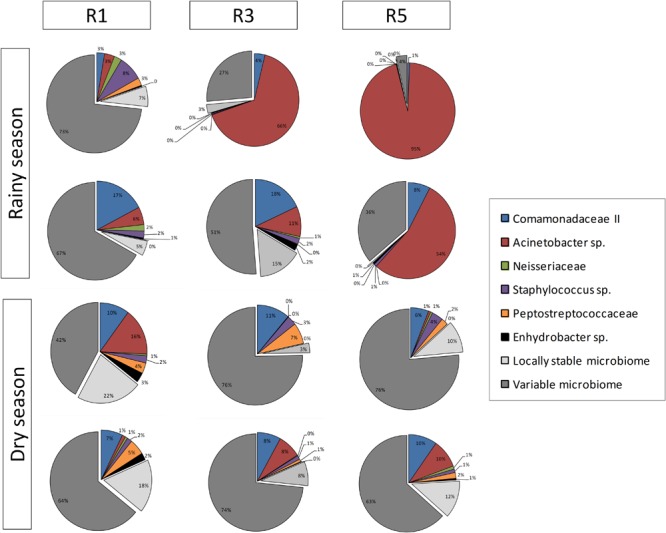
Overview of composition and relative abundance of *M. hispida* core bacteriome at different sampling locations (R1, R3, and R5) and over time (rainy and dry seasons) (*n* = 4).

The abundance of each core member varied among the different reefs at different sampling times (**Figure 3**). The following bacterial populations were identified as core members: *Acinetobacter* sp., Comamonadaceae II, Neisseriaceae, *Staphylococcus* sp., *Enhydrobacter* sp., and Peptostreptococcaceae. In general, the bacteriome core was dominated by *Acinetobacter* sp. and Comamonadaceae II. Neisseriaceae was the third most prevalent OTU at Reef 1 in rainy-season samples. Although differences were detected, the distribution of the core bacterial members was homogeneous during the dry season.

**FIGURE 4:**
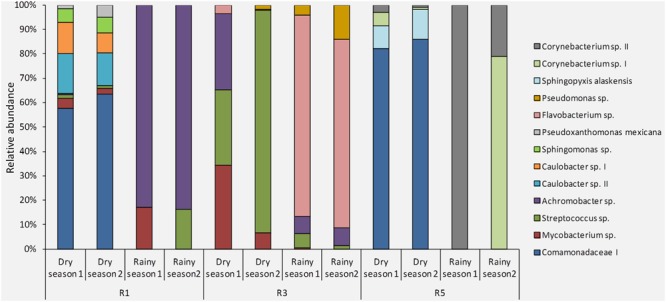
Composition and relative abundance of the site-stable bacterial populations (persistent OTUs found within each reef/sampling point) of *M. hispida* at different sampling locations (R1, R3, and R5) and over time (rainy and dry seasons) (*n* = 4).

Overall, the site-stable bacterial populations from dry-season samples were more similar to each other (**Figure 4**). The site-stable bacterial population from Reef 1 was composed of 9 different OTUs (Comamonadaceae I, *Streptococcus* sp., *Mycobacterium* sp., *Pseudoxanthomonas mexicana, Sphingomonas* sp., *Caulobacter* spp. I and II, *Streptococcus* sp., *Achromobacter* sp.). The site-stable bacterial populations associated with Reef 3 were represented by five OTUs (*Achromobacter* sp., *Streptococcus* sp., *Mycobacterium* sp., *Pseudomonas* sp., and *Flavobacterium* sp.). Finally, the site-stable bacterial populations from Reef 5 were composed of four OTUs (Comamonadaceae I, *Corynebacterium* spp. I and II, and *Sphingopyxis alaskensis*) (**Figure 4** and **Table 1**).

Based on a Venn diagram, most of the site-stable bacteria were exclusive to each reef. However, Comamonadaceae I occurred at Reefs 1 and 5, and also *Achromobacter* sp., *Mycobacterium* sp., and *Streptococcus* sp. occurred at Reefs 1 and 3 (**Supplementary Figure S7**).

**FIGURE 5:**
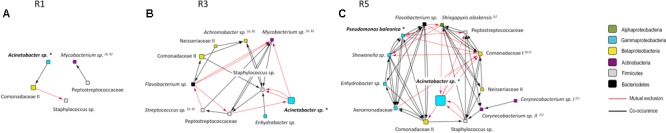
Network analysis of interactions among the 150 most abundant bacterial OTUs at **(A)** Reef 1, **(B)** Reef 3, and **(C)** Reef 5. The size of the nodes reflects the relative abundance of the genus in the entire data set, and the nodes are sorted and colored by phylum, except for the Proteobacteria, shown at class level. The core bacteriome members found in this study are highlighted in gray. ^∗^Indicates the vertically transferred members of the core bacteriome (Leite et al., 2017). ^a^Indicates the R1 site-stable bacterial populations (persistent OTUs found at each reef/sampling point). ^b^Indicates the R3 site-stable bacterial populations. ^c^Indicates the R5 site-stable bacterial populations.

Co-occurrence networks were generated for each reef individually, based on their 150 most abundant OTUs (**Figure 5**). The overall topological properties of these networks were also calculated in order to distinguish the OTU correlations at the different reefs (**Table 2**). For the number of nodes, edges and correlations (positive and negative), Reef 5 showed the highest values and Reef 1 the lowest (**Table 2** and **Figure 5**), indicating that a more-complex network topology existed at Reef 5 than at Reefs 3 and 1 (**Table 2**).

**Table 2:** General topological properties of network analysis using the 150 most abundant OTUs of Reefs 1, 3, and 5.

Network metrics	R1	R3	R5
Number of nodes^a^	5	10	14
Total number of edges^b^	3	22	57
Number of positive correlation^c^	1	14	40
Number of negative correlations^d^	2	8	13

*^*a*^Number of OTUs with at least one correlation. Correlation magnitude* > *0.5 (p < 0.05). ^*b*^Number of connection between the nodes. ^*c*^Correlation magnitude* > *0.5 (p < 0.05). ^*d*^Correlation magnitude < -0.5 (p < 0.05). OTUs, operational taxonomic units.*

Bacterial core members were easily found in network interactions from Reefs 1, 3, and 5, including *Acinetobacter* sp., Comamonadaceae II, *Staphylococcus* sp., Peptostreptococcaceae, and *Enhydrobacter* sp. (**Figure 5**). Some site-stable bacterial populations (persistent OTUs found at each reef/sampling point) were also observed, such as *Mycobacterium* sp., *Streptococcus* sp., *Achromobacter* sp., Comamonadaceae I, *Corynebacterium* spp. I and II, and *Sphingopyxis alaskensis*. Close relationships among *Acinetobacter* sp., Comamonadaceae II, and *Staphylococcus* sp. were observed in all network analyses (**Figure 5**).

## Discussion

The bacterial diversity associated with the surrounding seawater was higher than in the coral bacteriome. Additionally, the two bacteriomes (coral and seawater) showed significantly different profiles, as previously reported for different coral species (Rosenberg et al., 2007a,b; Castro et al., 2010; Rojo, 2010; Garcia et al., 2013) and also specifically for the *M. hispida* bacteriome (Leite et al., 2017, 2018).

Overall, the bacterial communities associated with *M. hispida*, sampled on different reefs for nearly 1 year, were extremely dynamic. In particular, six members composed the core bacteriome of *M. hispida*, considering all sampled reefs and times (*Acinetobacter* sp., Comamonadaceae II, Neisseriaceae, *Staphylococcus* sp., *Enhydrobacter* sp., and Peptostreptococcaceae), and comprised ∼20–96% of the total bacterial community members. In some cases, *Acinetobacter* sp. appeared as the predominant member, such as during the rainy season at Reef 5. This genus has been described as being vertically transmitted from parent to offspring in *M. hispida* (Leite et al., 2017), which suggests a potential key role of this group for the holobiont. On the other hand, the genus *Acinetobacter* has been detected in a wide range of hosts (Wegley et al., 2007; Shnit-Orland and Kushmaro, 2009; Sweet et al., 2013; Li Y. et al., 2014; Badhai et al., 2016; Meyer et al., 2016; Zhou et al., 2016; Leite et al., 2017), including many tropical corals (Wegley et al., 2007; Sweet et al., 2013; Leite et al., 2017), where this genus has been considered as an effective first line of defense (Santos et al., 2015; Leite et al., 2017) and also as potential pathogens (Shnit-Orland and Kushmaro, 2009; Sweet et al., 2013; Li J. et al., 2014; Meyer et al., 2016).

**FIGURE 6:**
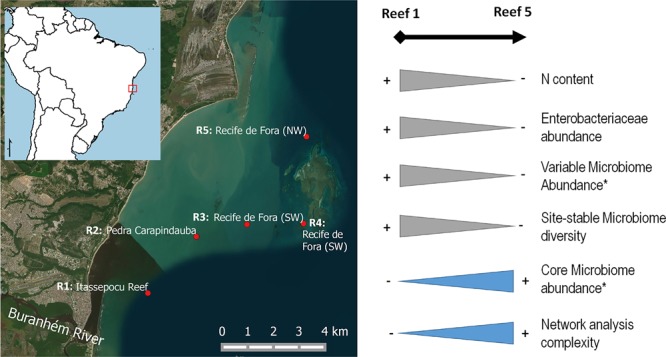
Overview of the different gradients found at different sampling locations, Reef 1 (R1), Reef 3 (R3), and Reef 5 (R5). ^∗^Using only the gradients observed in the rainy seasons.

Several studies on coral bacteriomes have revealed that these organisms can quickly respond when they are faced with environmental impacts, such as pollution and climate change (Kelly et al., 2014; Santos et al., 2014, 2015; Pantos et al., 2015; Roder et al., 2015; McCauley et al., 2016; Sharp et al., 2017; van de Water et al., 2017; Wang et al., 2018). Similarly, our main findings indicated that the reef farthest from the Buranhém River mouth (Reef 5) showed a higher proportion of members of the bacterial-core microbiome, especially in the rainy seasons. Reef 5 was also the location with the lowest concentrations of pollution proxies (N content and Enterobacteriaceae abundance), suggesting that members of the bacterial core could be more abundant under environmental conditions that are more similar to less-polluted areas, compared to more-polluted areas. Inversely, higher relative abundances of the variable bacterial populations seemed to be related to more-polluted areas, also during the rainy seasons when the river influence probably increased (**Figures 2, 6**). In addition, the site-stable bacterial diversity was proportionally higher at the nearshore Reef 1 (closest to the Buranhém River mouth) during the rainy seasons, suggesting a local bacterial adaptation due to environmental changes and impacts. Our data also indicated a higher diversity of OTUs representing the site-stable bacterial populations, which could be related to the higher abundance of total coliforms and higher N content (proxies for sewage or organic pollution) found at Reef 1.

Additionally, bacterial members of the core and site-stable bacterial populations were also observed in co-occurrence networks. Network analyses represent the complexity of ecological relationships (whether there are co-occurring relationships or mutual exclusions) among the microbial players (Barberán et al., 2012; Faust and Raes, 2012). In general, the greater the stability of the microbiome in a given environment, the greater the complexity of these relationships, since these organisms and environments are less susceptible to microbial invasion (van Elsas et al., 2012; Mallon et al., 2015), or switching (acquisition of microbes from seawater) (Webster and Reusch, 2017). Our data indicated a clear and significant increasing gradient of complexity of the network relationships, from Reef 1 (least-complex topology) to Reef 5 (most-complex topology) (**Figure 5**), considering all sampling times, the same gradient as observed for the proxies of organic pollution. This lack of complexity of the network relationships on the reefs that were more exposed to organic pollution, leading to a more susceptible bacteriome (Mallon et al., 2015), could explain why chronic nutrient enrichment seems to render some coral OTUs more susceptible to diseases and bleaching (Vega Thurber et al., 2014).

Recently, Zaneveld et al. (2017) proposed the Anna Karenina Principle (AKP) for host-associated bacteriomes. The AKP hypothesizes that some stressors have stochastic, rather than deterministic, effects on the host-associated bacteriome. Our data concord with the AKP, since the reefs offshore, and therefore less “polluted” areas, had a more-complex network and high dominance of the bacterial-core members, in terms of relative abundance (around 96% in some cases), especially during rainy seasons, thus resulting in a more homogeneous and well-established microbiome. On the other hand, the coral bacteriome recovered from nearshore samples, also during rainy seasons, showed high variation among replicates in their associated bacterial populations, which could be related to the chronic increase in the contents of nutrients such as N. This could lead to the possible loss of putatively important players, such as *Pseudomonas* sp. This genus was previously described as a member of the *M. hispida* bacterial core (Leite et al., 2017, 2018), and was also recovered as a member of the bacterial core of Reefs 3 and 5. We suggest that the influence of the river mouth can generate a type of chronic anthropogenic contamination, not only represented by the loss of the genus *Pseudomonas*, but also based on the relatively high proportion of site-stable bacterial populations at Reef 1, compared to the other reefs.

A balance between stable and variable bacteriomes is often observed in communities subject to environmental impacts (Kelly et al., 2014; Pantos et al., 2015; Roder et al., 2015; Hernandez-Agreda et al., 2016; McCauley et al., 2016; Mustafa et al., 2016; van de Water et al., 2017). The microbial core is also dynamic in this respect, since the core microorganisms themselves may be more or less frequent (or even disappear) depending on environmental conditions (McCauley et al., 2016; Leite et al., 2017, 2018). Here, we also detected a stable local-microbiota that was as dynamic as the core. Changes in the bacterial structure or composition were correlated primarily with changes in the relative abundance of common bacterial members. This pattern is aligned with recent assumptions that bacteriomes can respond to stress through shifts in the proportional abundances of the community members, which can also lead to the disappearance or appearance of specific taxa (Sharp et al., 2017; Webster and Reusch, 2017). Enrichment of Enterobacteria OTUs (total coliforms), a proxy for human-sourced sewage pollution, was apparent in the coral samples at Reef 1, as was a significantly higher N content in the seawater at that site. These may be some of the causes of the observed shifts in the site-stable bacterial populations, and in the dynamic core and variable relative abundances. If such environmentally driven shifts in the bacteriome persist, they may lead to a local adaptation of the bacteriome, which could also be transferred to the offspring (Webster and Reusch, 2017).

Taken together, these results indicate that the Reef 5 environment is less polluted than at Reef 1, reflected not only in the higher dominance of the bacterial core, but also the greater complexity of the bacteriome network at the former. Reef 1, in turn, serves as an example of an impacted cliff reef, with a low proportion of the bacterial core and significantly less-complex bacterial relationships. These analyses also indicate that the coral-associated bacterial core and coral bacteriome networks can be used as additional targets for evaluation, as proxies for anthropogenic pollution.

## Data Accessibility

The raw data from each sample are available in the NCBI Sequence Read Archive (SRA) under the Accession Number (SAMN08391548–SAMN08391636).

## Author Contributions

RP, DL, and EC: study conception and design. DL, EC, and CC: acquisition and identification of coral samples. DL, EC (biological), JM, and AB (chemical): acquisition of data (experimental development). RP, DL, JE, and JS: analyses and interpretation of data. RP and DL: drafting of the manuscript. RP, JS, and CC: financial support. All authors: critical revision of the manuscript.

## Conflict of Interest Statement

The authors declare that the research was conducted in the absence of any commercial or financial relationships that could be construed as a potential conflict of interest. The reviewer DTS and handling Editor declared their shared affiliation.

## Footnotes

1 https://qiime2.org/

## Supplementary Material

FIGURE S1 Scheme demonstrating the experimental strategy used in this study. The horizontal arrow indicates the timeline. The vertical arrow in gray indicates the river’s influence on the reefs.

FIGURE S2 Non-metric multidimensional scaling (NMS) based on the quantitative values of the matrix profiles of the PCR-DGGE profile for the seawater bacterial communities in the rainy **(A,B)** and dry **(C,D)** seasons (*n* = 4).

FIGURE S3 Non-metric multidimensional scaling based on the quantitative values of the matrix profiles of the PCR-DGGE profile for the bacterial communities associated with the coral *Mussismilia hispida* in the rainy **(A,B)** and dry **(C,D)** seasons (*n* = 4).

FIGURE S4 (A) Principal coordinates analysis (PCoA) (weighted UniFrac) based on the distance matrix for operational taxonomic units (OTUs), showing differences between seawater and *M. hispida* bacteriomes (PERMANOVA, pseudo-F: 204.47, *p* < 0.001), and **(B)** Richness of seawater and *M. hispida* bacteriome based on phylogenetic distance (Tukey test, *p* < 0.001).

FIGURE S5 Rarefaction curves from bacterial communities of *M. hispida* (considering OTU level) at different sampling locations (R1, R3, and R5) and over time (rainy and dry seasons).

FIGURE S6 (A) Absolute (mean standard deviation) abundance of Enterobacteriaceae (represented by three different OTUs) in *M. hispida* bacteriomes at different sampling locations (R1, R3, and R5) (*n* = 4). Different letters indicate significant difference between samples (Mann–Whitney test, *p* < 0.05). **(B)** Correlation plot showing the functional correlation between Enterobacteriaceae OTUs from *M. hispida* bacteriomes and seawater N contents. Filled diamonds indicate the variables.

FIGURE S7 Venn diagram displaying the degree of overlap of bacterial OTUs between the site-stable bacterial populations (persistent OTUs found within each reef/sampling point) from bacteriomes of Reefs 1, 3, and 5. The vertically transferred core bacteriome members (Leite et al., 2017) are highlighted.

TABLE S1 Environmental conditions at the sampling locations (*n* = 4).
