# Supplementary figures and images for: Coral Bacterial-Core Abundance and Network Complexity as Proxies for Anthropogenic Pollution
Source: Front Microbiol. 2018 Apr 27;9:833. doi: 10.3389/fmicb.2018.00833 (PMC5934943; doi:10.3389/fmicb.2018.00833)

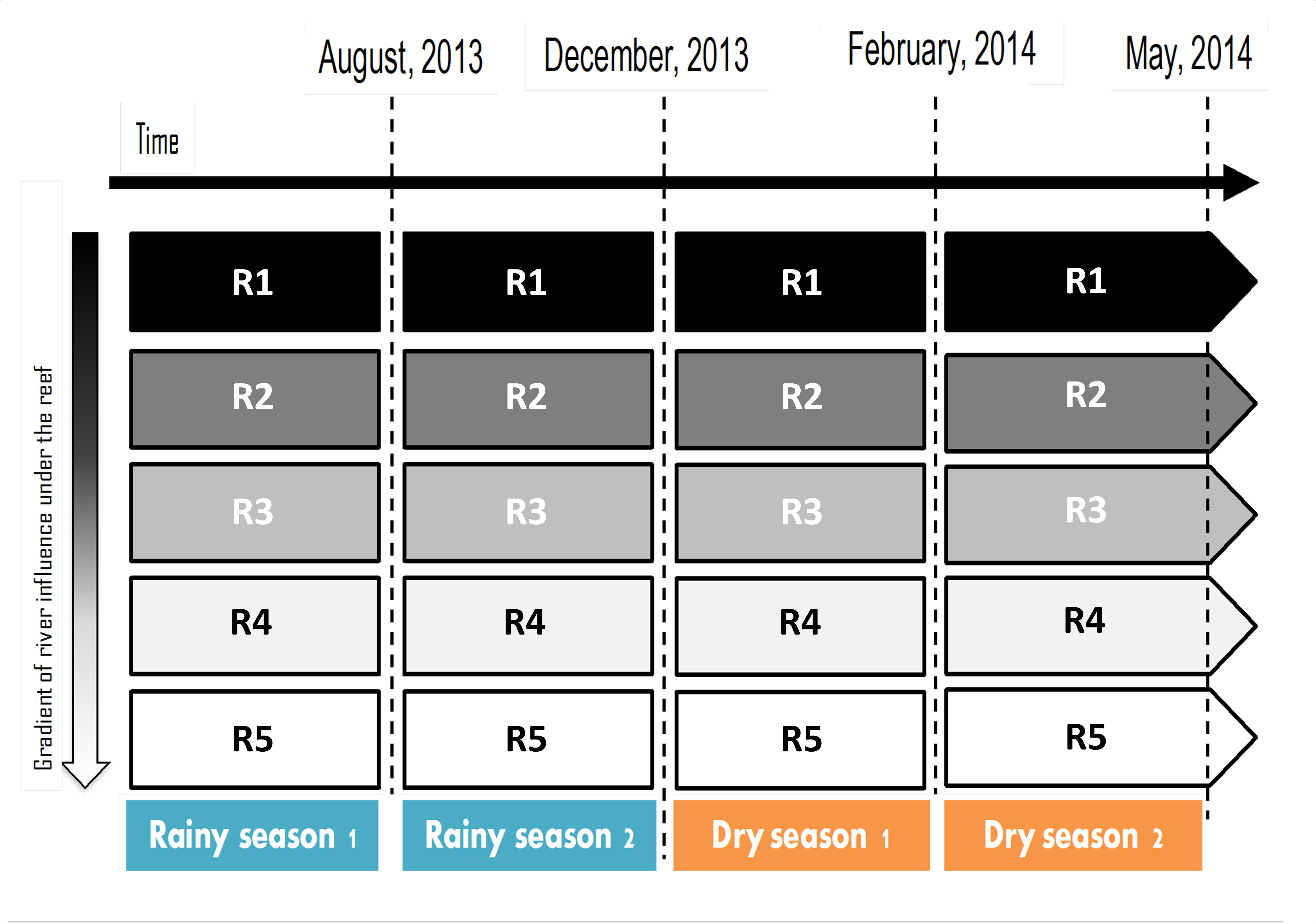

Supplement: FIGURE S1 — Scheme demonstrating the experimental strategy used in this study. The horizontal arrow indicates the timeline. The vertical arrow in gray indicates the river’s influence on the reefs. [file Image_1.TIF]

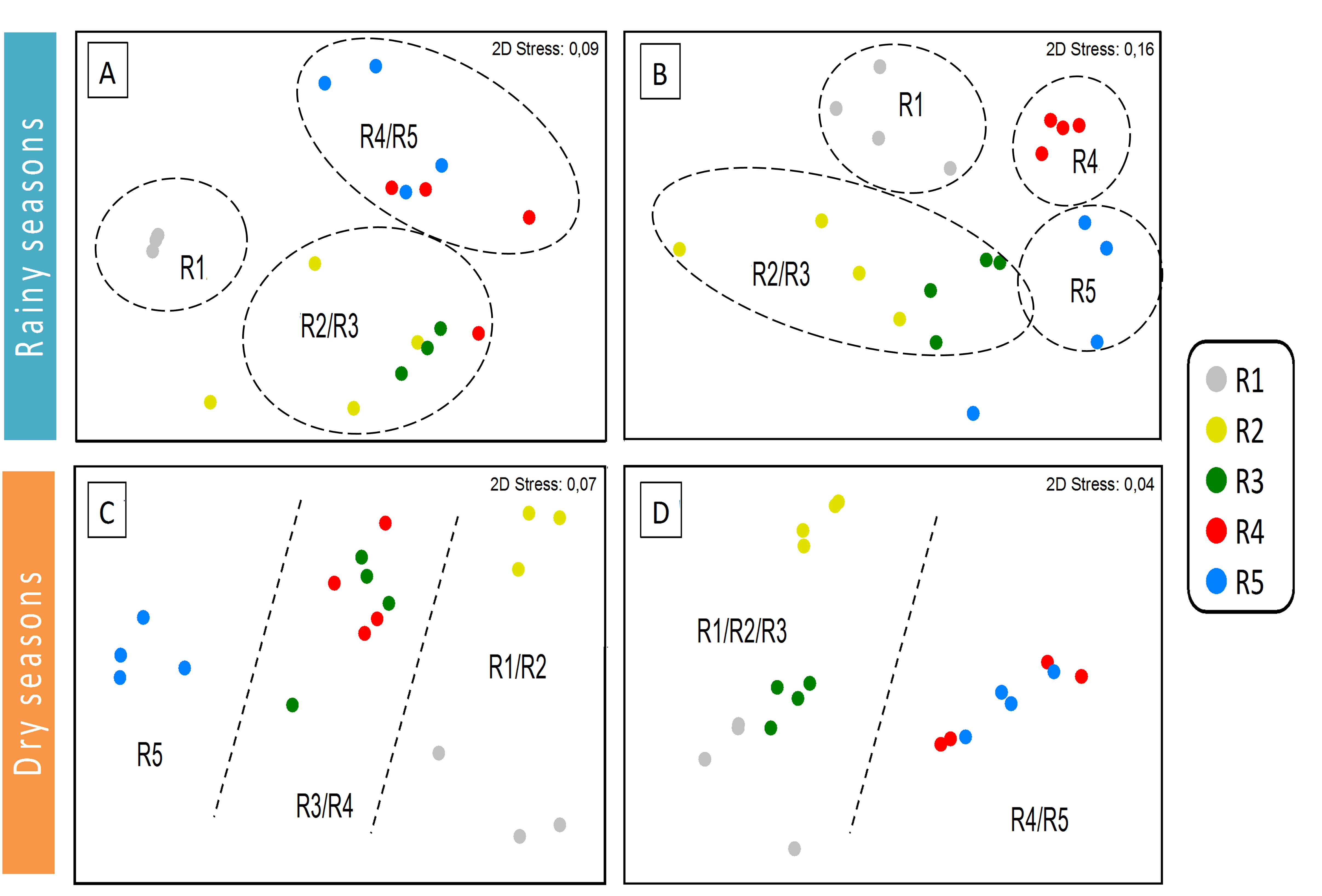

Supplement: FIGURE S2 — Non-metric multidimensional scaling (NMS) based on the quantitative values of the matrix profiles of the PCR-DGGE profile for the seawater bacterial communities in the rainy (A,B) and dry (C,D) seasons (n = 4). [file Image_2.TIF]

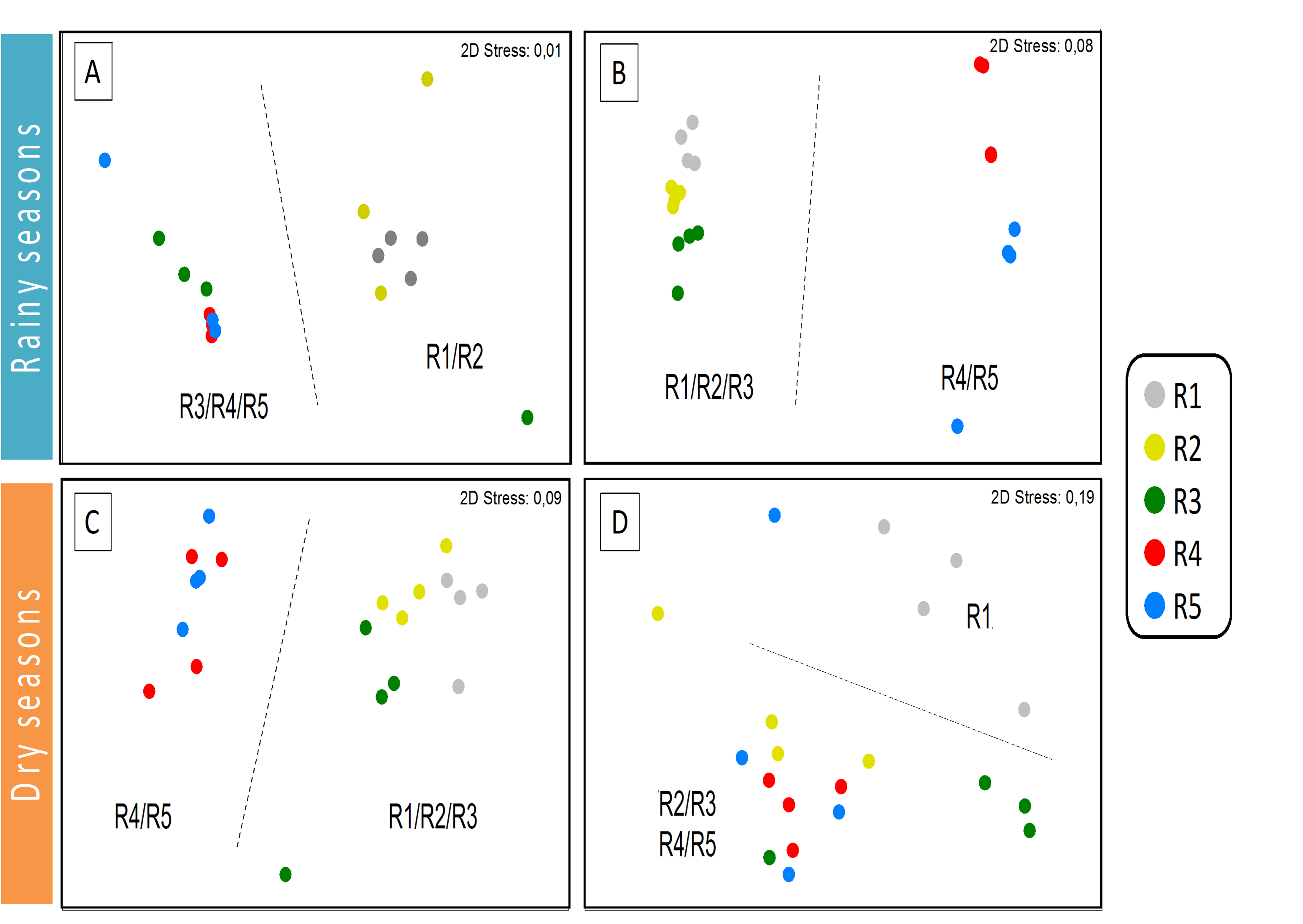

Supplement: FIGURE S3 — Non-metric multidimensional scaling based on the quantitative values of the matrix profiles of the PCR-DGGE profile for the bacterial communities associated with the coral Mussismilia hispida in the rainy (A,B) and dry (C,D) seasons (n = 4). [file Image_3.TIF]

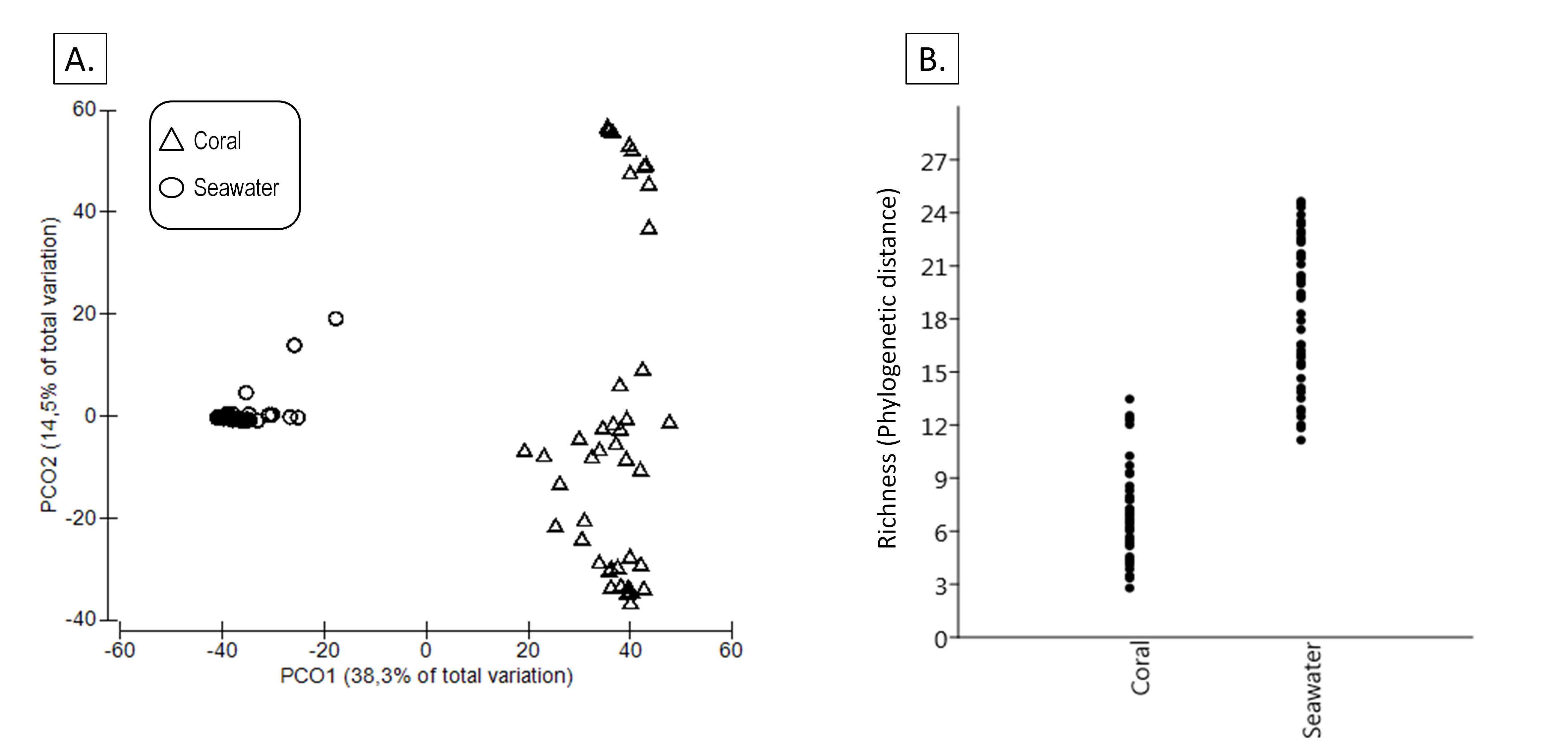

Supplement: FIGURE S4 — (A) Principal coordinates analysis (PCoA) (weighted UniFrac) based on the distance matrix for operational taxonomic units (OTUs), showing differences between seawater and M. hispida bacteriomes (PERMANOVA, pseudo-F: 204.47, p < 0.001), and (B) Richness of seawater and M. hispida bacteriome based on phylogenetic distance (Tukey test, p < 0.001). [file Image_4.TIF]

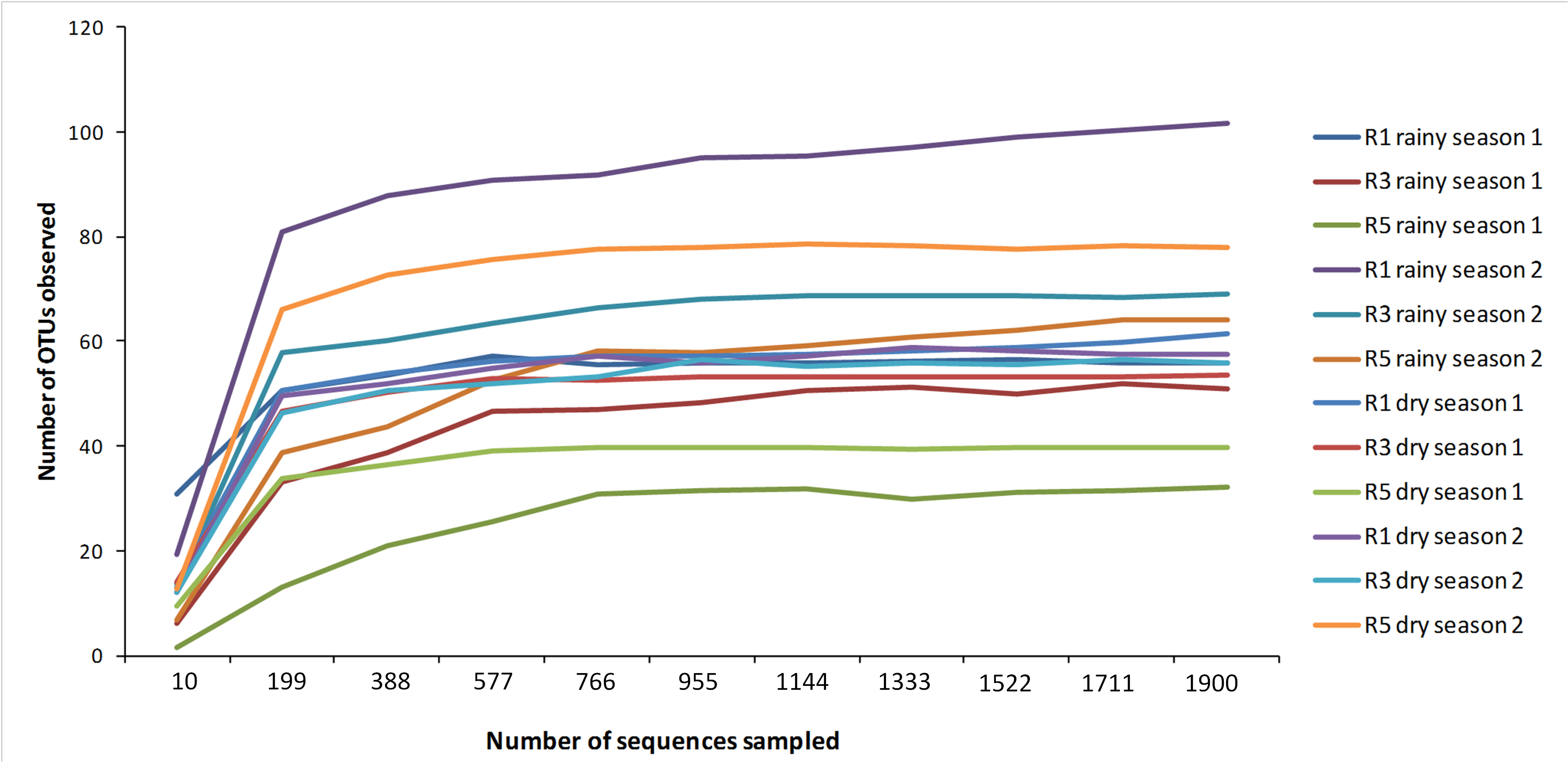

Supplement: FIGURE S5 — Rarefaction curves from bacterial communities of M. hispida (considering OTU level) at different sampling locations (R1, R3, and R5) and over time (rainy and dry seasons). [file Image_5.TIFF]

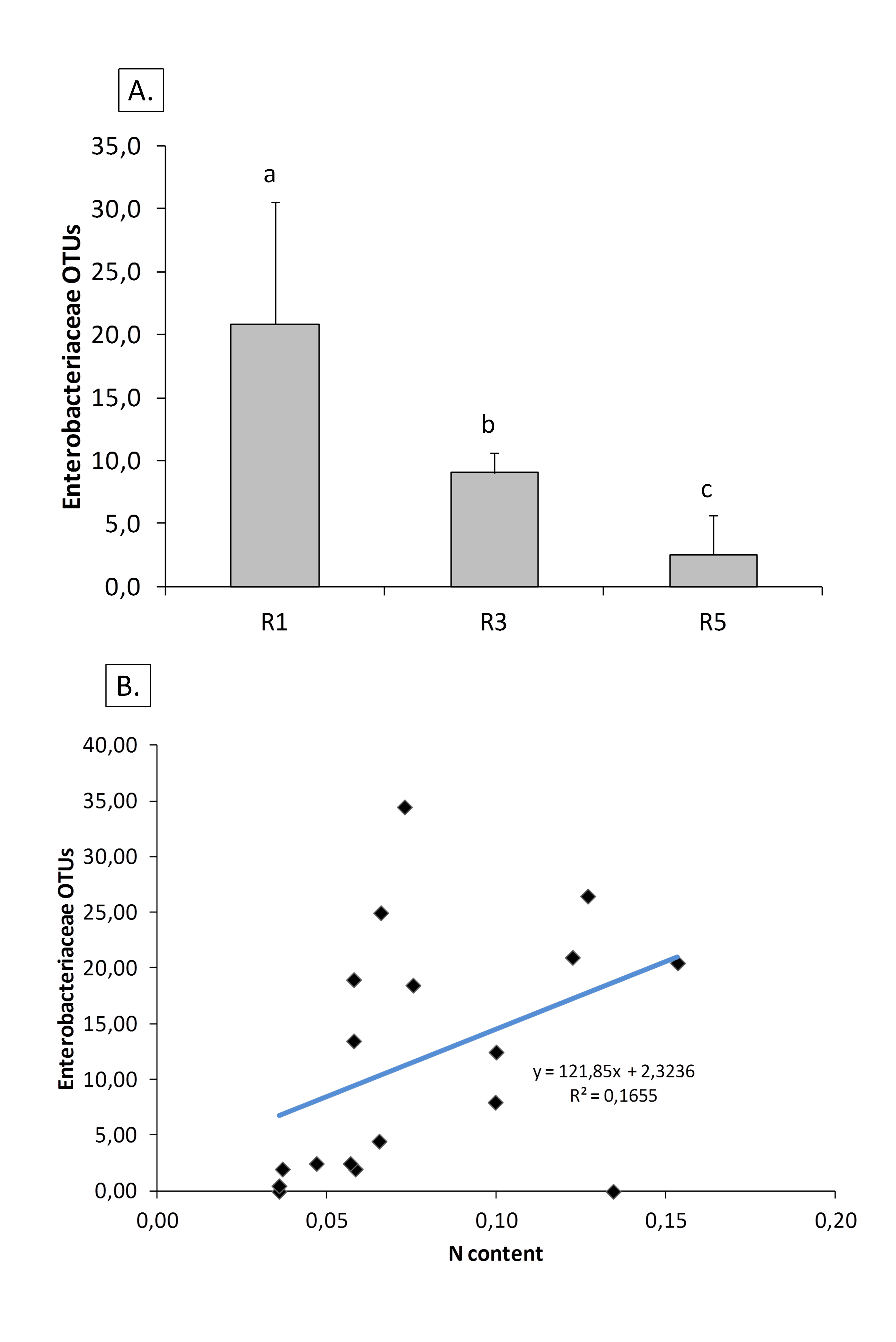

Supplement: FIGURE S6 — (A) Absolute (mean standard deviation) abundance of Enterobacteriaceae (represented by three different OTUs) in M. hispida bacteriomes at different sampling locations (R1, R3, and R5) (n = 4). Different letters indicate significant difference between samples (Mann–Whitney test, p < 0.05). (B) Correlation plot showing the functional correlation between Enterobacteriaceae OTUs from M. hispida bacteriomes and seawater N contents. Filled diamonds indicate the variables. [file Image_6.TIF]

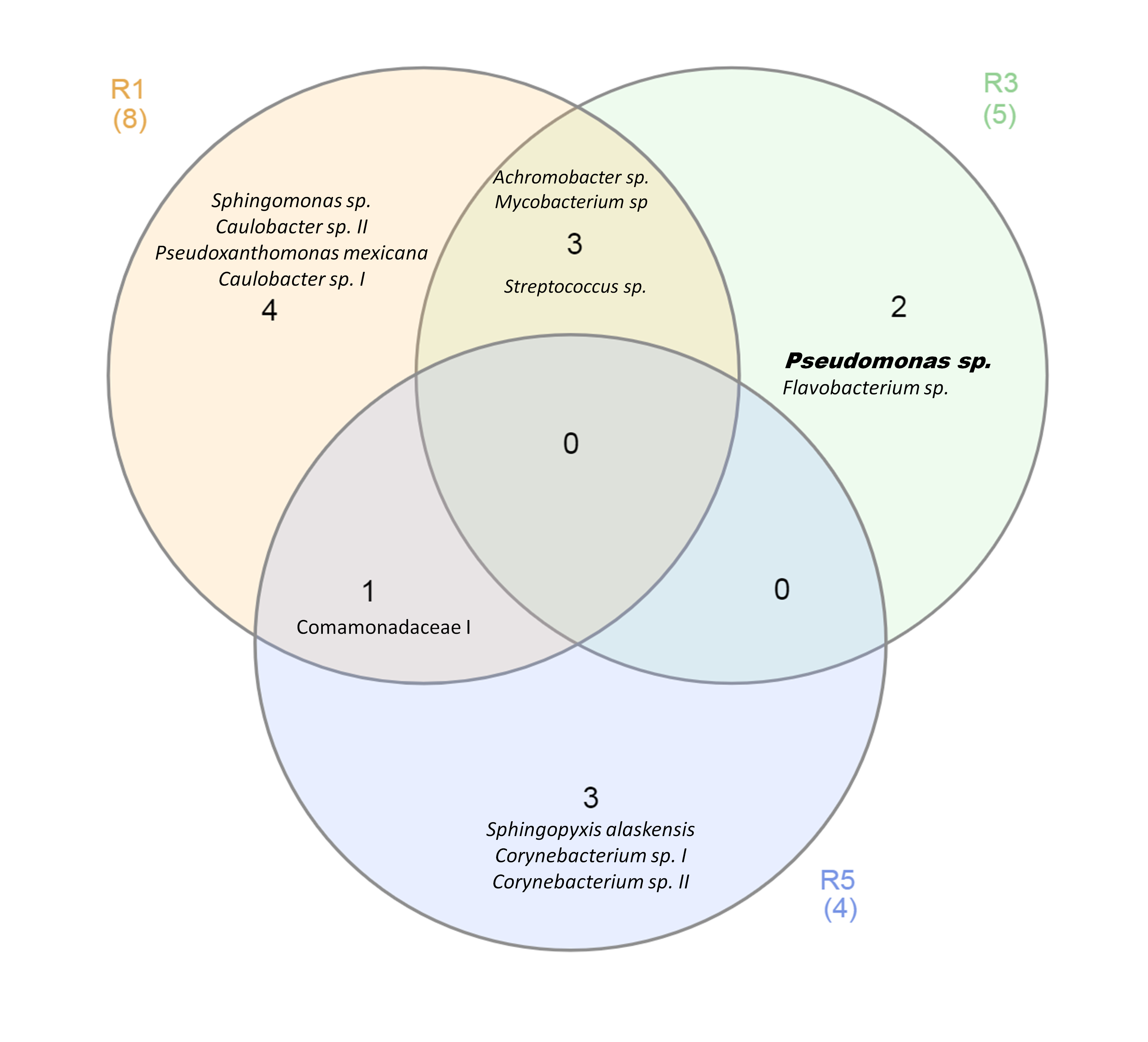

Supplement: FIGURE S7 — Venn diagram displaying the degree of overlap of bacterial OTUs between the site-stable bacterial populations (persistent OTUs found within each reef/sampling point) from bacteriomes of Reefs 1, 3, and 5. The vertically transferred core bacteriome members (Leite et al., 2017) are highlighted. [file Image_7.TIF]
