# Supplementary material for: Coral Bacterial-Core Abundance and Network Complexity as Proxies for Anthropogenic Pollution
Source: Front Microbiol. 2018 Apr 27;9:833. doi: 10.3389/fmicb.2018.00833 (PMC5934943; doi:10.3389/fmicb.2018.00833)
Supplement: TABLE S1 — Environmental conditions at the sampling locations (n = 4). [file Table_1.docx]

**Supplementary Table**

**Table S1.** Environmental conditions at the sampling locations (n = 4).

|  |  | **Sediment** | | | | | | | | |  | **Seawater** | | | | | |
| --- | --- | --- | --- | --- | --- | --- | --- | --- | --- | --- | --- | --- | --- | --- | --- | --- | --- |
| **Site** | **Period** | **N (mg/L)** | | | **C (mg/L)** | | | **Carbonate (mg/L)** | | |  | **DOC (mg/L)** | | | **Na (mM)** | **K (mM)** | **Ca (mM)** |
| R1 | Rainy season 1 |  | - |  |  | - |  |  | - |  |  | 0.85 | ± | 0.05 | 618.23 | 9.38 | 11.22 |
|  | Rainy season 2 | 0.06 | ± | 0.01 | 2.57 | ± | 1.04 | 89.18 | ± | 7.65 |  | 1.23 | ± | 0.01 | - | - | - |
|  | Dry season 1 | 0.10 | ± | 0.01 | 2.33 | ± | 0.20 | 75.68 | ± | 2.45 |  | 1.23 | ± | 0.04 | 615.00 | 5.12 | 7.27 |
|  | Dry season 2 | 0.09 | ± | 0.01 | 2.59 | ± | 0.07 | 81.40 | ± | 1.57 |  | 3.12 | ± | 1.24 | 686.96 | 11.94 | 11.64 |
| R2 | Rainy season 1 | 0.04 | ± | 0.00 | 2.54 | ± | 0.29 | 34.60 | ± | 3.82 |  | 0.51 | ± | 0.12 | 615.67 | 10.23 | 7.07 |
|  | Rainy season 2 | 0.07 | ± | 0.01 | 1.05 | ± | 0.42 | 59.99 | ± | 6.36 |  | 7.84 | ± | 0.52 | 680.65 | 11.94 | 12.88 |
|  | Dry season 1 | 0.04 | ± | 0.00 | 0.80 | ± | 0.28 | 51.33 | ± | 0.47 |  | 2.01 | ± | 0.50 | 542.41 | 5.12 | 5.40 |
|  | Dry season 2 | 0.14 | ± | 0.03 | 2.76 | ± | 0.19 | 72.48 | ± | 11.20 |  | 3.53 | ± | 1.12 | 646.50 | 5.12 | 6.65 |
| R3 | Rainy season 1 | 0.16 | ± | 0.01 | 2.65 | ± | 0.12 | 66.60 | ± | 3.16 |  | 0.69 | ± | 0.18 | 689.00 | 5.12 | 7.48 |
|  | Rainy season 2 | 0.03 | ± | 0.00 | 5.36 | ± | 2.83 | 59.41 | ± | 27.39 |  | 8.01 | ± | 0.38 | 530.89 | 7.25 | 10.81 |
|  | Dry season 1 | 0.10 | ± | 0.03 | 2.00 | ± | 0.53 | 68.90 | ± | 1.65 |  | 1.72 | ± | 0.03 | 599.26 | 6.39 | 14.13 |
|  | Dry season 2 | 0.10 | ± | 0.00 | 1.36 | ± | 0.16 | 55.62 | ± | 1.04 |  | 3.82 | ± | 0.38 | 562.17 | 7.67 | 16.63 |
| R4 | Rainy season 1 | 0.04 | ± | 0.00 | 3.23 | ± | 0.19 | 95.04 | ± | 1.58 |  | 0.56 | ± | 0.42 | 636.04 | 6.82 | 7.90 |
|  | Rainy season 2 | 0.06 | ± | 0.01 | 2.31 | ± | 0.64 | 88.98 | ± | 3.37 |  | 1.02 | ± | 0.01 | 645.50 | 6.82 | 9.14 |
|  | Dry season 1 | 0.04 | ± | 0.00 | 4.86 | ± | 0.27 | 59.34 | ± | 2.11 |  | 4.77 | ± | 1.73 | 570.32 | 8.10 | 11.43 |
|  | Dry season 2 | 0.15 | ± | 0.07 | 1.39 | ± | 0.37 | 64.25 | ± | 2.39 |  | 4.14 | ± | 0.89 | 608.64 | 5.97 | 10.81 |
| R5 | Rainy season 1 | 0.06 | ± | 0.02 | 2.57 | ± | 0.61 | 83.16 | ± | 6.16 |  | 0.49 | ± | 0.15 | 609.65 | 8.53 | 8.31 |
|  | Rainy season 2 | 0.09 | ± | 0.02 | 3.73 | ± | 0.34 | 83.49 | ± | 3.57 |  | 5.84 | ± | 3.09 | 595.88 | 10.23 | 7.48 |
|  | Dry season 1 | 0.08 | ± | 0.01 | 2.94 | ± | 0.01 | 85.87 | ± | 1.33 |  | 6.28 | ± | 0.85 | 655.78 | 7.67 | 10.60 |
|  | Dry season 2 | 0.08 | ± | 0.02 | 5.40 | ± | 0.25 | 92.69 | ± | 2.95 |  | 2.58 | ± | 0.62 | 620.74 | 10.23 | 8.10 |

Values for N, C, carbonate and DOC are given as mean ± standard deviation. Na, K and C ions are given as mean.
